# A Novel Inflammation-Based Prognostic Score: The Fibrinogen/Albumin Ratio Predicts Prognoses of Patients after Curative Resection for Hepatocellular Carcinoma

**DOI:** 10.1155/2018/4925498

**Published:** 2018-05-22

**Authors:** Qiaodong Xu, Yongcong Yan, Songgang Gu, Kai Mao, Jianlong Zhang, Pinbo Huang, Zhenyu Zhou, Zheng Chen, Shaodong Zheng, Jiahong Liang, Zhihua Lin, Jie Wang, Jiang Yan, Zhiyu Xiao

**Affiliations:** ^1^Guangdong Province Key Laboratory of Malignant Tumor Epigenetics and Gene Regulation, Research Center of Medicine, Sun Yat-sen Memorial Hospital, No. 107 Yanjiang Western Road, Guangzhou 510120, China; ^2^Department of Hepatobiliary Surgery, Sun Yat-sen Memorial Hospital, No. 33 Yingfeng Road, Guangzhou 510289, China; ^3^Department of Biliary-Pancreatic Minimally Invasive Surgery, The First Affiliated Hospital of Shantou University Medical College, No. 57 Changping Road, Shantou 515041, China

## Abstract

**Background:**

Inflammation is an important hallmark of cancer. Fibrinogen and albumin are both vital factors in systemic inflammation. This study investigated the prognostic value of the fibrinogen/albumin ratio in HCC patients who underwent curative resection.

**Methods:**

HCC patients (*n* = 151) who underwent curative resection were evaluated retrospectively. The optimal cutoff value for the fibrinogen/albumin ratio was selected by receiver operating characteristic (ROC) curve analysis. Correlations between preoperative fibrinogen/albumin ratios and clinicopathologic characteristics were analyzed by *χ* ^2^ test. The area under the receiver operating characteristic curve (AUC) was calculated to compare the prognostic value of the fibrinogen/albumin ratio with other prognostic scores (neutrophil to lymphocyte ratio (NLR), platelet to lymphocyte ratio (PLR), and albumin-bilirubin (ALBI) score). The overall survival (OS) and time to recurrence (TTR) were assessed by the log-rank test and the Cox proportional hazard regression model.

**Results:**

An optimal cutoff value of the preoperative fibrinogen/albumin ratio (0.062) was determined for 151 patients who underwent curative resection for HCC via a ROC curve analysis. Fibrinogen/albumin ratio > 0.062 was significantly associated with microvascular invasion, an advanced BCLC stage, and ALBI grade. Multivariate analyses revealed that fibrinogen/albumin ratio was an independent predictor for OS (*P* = 0.003) and TTR (*P* = 0.035). The prognostic ability of fibrinogen/albumin ratio was comparable to other prognostic scores (NLR, PLR, and ALBI score) by AUC analysis. Patients with a fibrinogen/albumin ratio > 0.062 had lower 1-, 3-, and 5-year OS rates (66.0%, 41.8%, and 28.2% versus 81.9%, 69.3%, and 56.1%, resp., *P* < 0.001) and higher 1-, 3-, and 5-year recurrence rates (60.9%, 79.2%, and 90.5% versus 49.5%, 69.1%, and 77.1%, resp., *P* = 0.008) compared with patients with fibrinogen/albumin ratio ≤ 0.062.

**Conclusion:**

The preoperative fibrinogen/albumin ratio is an effective prognostic factor for HCC patients who underwent curative resection. An elevated fibrinogen/albumin ratio significantly correlates with poorer survival and a higher risk of recurrence in HCC patients.

## 1. Introduction

Hepatocellular carcinoma (HCC) is the second leading cause of cancer-related deaths and the sixth most prevalent cancer worldwide [1]. Currently, surgery remains the main treatment for patients with HCC. Although surgical treatments have markedly improved the overall survival of HCC patients, the long-term survival rate is still unsatisfactory. Approximately 60% of patients experience recurrence or distant metastasis within 5 years, even after curative resection [2]. Some prognostic factors, including microvascular invasion, tumor-related factors, the Child–Pugh classification, and albumin-bilirubin (ALBI) score, have been reported as prognostic indicators in HCC patients who underwent hepatectomy [3–5]. However, effective prognostic factors are still absent, especially serum biomarkers.

Inflammation is an important hallmark of cancer [6]. Numerous clinical and experimental studies have convincingly supported the concept that inflammation is an important component of tumor progression [7, 8]. Recently, inflammation-based index and scoring systems, such as the neutrophil-to-lymphocyte ratio (NLR), platelet-to-lymphocyte ratio (PLR), C-reactive protein (CRP)/albumin (Alb) ratio, Glasgow Prognostic Score (GPS), and modified GPS, have been reported as useful prognostic indicators in HCC [9–13].

Fibrinogen (Fib) is a glycoprotein synthesized by hepatocytes. It is produced in response to proinflammatory cytokines. Similar to CRP, fibrinogen belongs to the positive acute-phase-response group of proteins, which are characterized by elevated levels during systemic inflammation [14]. After being converted to fibrin, it plays a significant role in the progression of blood coagulation. It has been reported that plasma fibrinogen levels are predictive of a poor prognosis in various cancers, including HCC [15–19]. Albumin is produced by the liver and is considered a negative acute-phase protein. The reduction of albumin levels during inflammation is likely associated with the effect of cytokines, such as interleukin-6 (IL-6) and tumor necrosis factor-*α* (TNF-*α*). Serum albumin has been shown to have protective properties, such as maintaining physiological homeostasis, antioxidant activity, anti-inflammatory effects, and the prevention of apoptosis [20]. Several inflammation-based prognostic indexes (e.g., CRP/Alb ratio, GPS, and modified GPS), which have been reported to have prognostic value for HCC, take preoperative serum albumin levels into consideration.

Recently, it was reported that the Fib/Alb ratio correlated with patient prognosis in esophageal squamous cell carcinoma [21]. However, whether the Fib/Alb ratio is associated with the prognosis of HCC patients after curative resection has not been elucidated.

Herein, we performed a retrospective study to investigate whether the Fib/Alb ratio has prognostic value in patients undergoing curative resection for HCC.

## 2. Materials and Methods

### 2.1. Patients and Specimens

A retrospective study was conducted in a primary cohort that included a total of 181 patients who underwent curative resection of HCC (defined as the complete removal of the tumor without residual cancer) in the department of Hepatobiliary Surgery of Sun Yat-Sen Memorial Hospital between 2006 and 2010. Of these, 13 patients who showed clinical evidence of infection or other inflammatory conditions and hematological diseases were excluded, and 17 patients were excluded due to a lack of clinical data. There were no relevant drugs and interventions used that may have directly influenced peripheral hematological components. In total, 151 patients were finally enrolled and evaluated.

The diagnosis of each patient was pathologically confirmed. Patients did not have signs of distant metastases nor had they received anticancer therapies before surgery. The patients' characteristics, clinicopathological factors, and postoperative survival and recurrence rates were recorded. Tumor stages were determined according to the Barcelona Clinic Liver Cancer (BCLC) staging classification [22]. Tumor differentiation was graded based on the Edmondson grading system [23]. The study was approved by the Sun Yat-Sen Memorial Hospital Research Ethics Committee, and informed consent was obtained from all participants.

### 2.2. Fibrinogen/Albumin Ratio, NLR, PLR, and ALBI Score

In all patients included in the study, blood samples were collected and routine laboratory analyses of plasma fibrinogen and albumin levels were performed during routine workup to exclude coagulation disorders or the presence of acute infections before surgery or diagnostic interventions. The total bilirubin, white blood cell count, neutrophil, lymphocyte, platelet count, and *α*-fetoprotein level (AFP) were measured as well. The Fib/Alb ratio was calculated by dividing the plasma fibrinogen level by the albumin level. The NLR was calculated by dividing the neutrophil count by the lymphocyte count [10]. The PLR was calculated by dividing the platelet count by the lymphocyte count [10]. The ALBI score was calculated from the formula, ALBI score = (log_10_ bilirubin × 0.66) + (albumin × −0.085), where bilirubin is in *μ*mol/L and albumin in g/L. Specific cutoffs were then applied to generate three prognostic groups: ALBI score ≤ −2.60 (ALBI grade 1), >−2.60 to ≤−1.39 (ALBI grade 2), and ALBI score > −1.39 (ALBI grade 3) [24].

### 2.3. Treatment and Follow-Up

All patients were observed over a median observation time of 33.8 months (range, 1 to 86 months). Patients were monitored by examining serum alpha-fetoprotein (AFP) levels and performing an abdominal ultrasound every 2 months during the first year after the operation and every 3 to 4 months thereafter. Magnetic resonance imaging or computed tomography, together with chest radiographic examination, was performed every three to six months in the first two years and every six to twelve months thereafter. Upon suspicion of recurrence or metastasis, chest computed tomography, magnetic resonance imaging, and bone scintigraphy were performed for confirmation. Overall survival (OS) and time to recurrence (TTR) were considered as the primary endpoints. OS was defined as the interval between surgery and death or between surgery and the last follow-up time for surviving patients. TTR was defined as the interval between surgery and recurrence or between surgery and the last follow-up time for patients without recurrence.

### 2.4. Statistical Analysis

Statistical analyses were performed using SPSS for Windows (version 19.0). A receiver operating characteristic (ROC) curve analysis was performed to select the optimal cutoff value for the Fib/Alb ratio. The *χ* ^2^ test was used to compare categorical variables. Cumulative survival and recurrence rates were calculated using a Kaplan–Meier analysis and log-rank test. A Cox proportional hazard regression model was used to assess prognostic factors. Factors with a *P* < 0.05 in the univariate analysis were included in the multivariate analysis. The final multivariate analysis was performed using a forward stepwise procedure for variable selection. *P* < 0.05 was considered statistically significant.

## 3. Results

### 3.1. Patient Characteristics

The median age of the patients was 51 (range 22–78) years, 128 (84.8%) were males and 23 (15.2%) were females, and 132 (87.4%) were positive for hepatitis B surface antigen (HBs-Ag) and 19 (12.6%) were negative for HBs-Ag. The detailed clinical characteristics of the 151 patients are summarized in Table 1.

### 3.2. Determination of Optimal Cutoff Value of Fib/Alb Ratio

**Figure 1:**
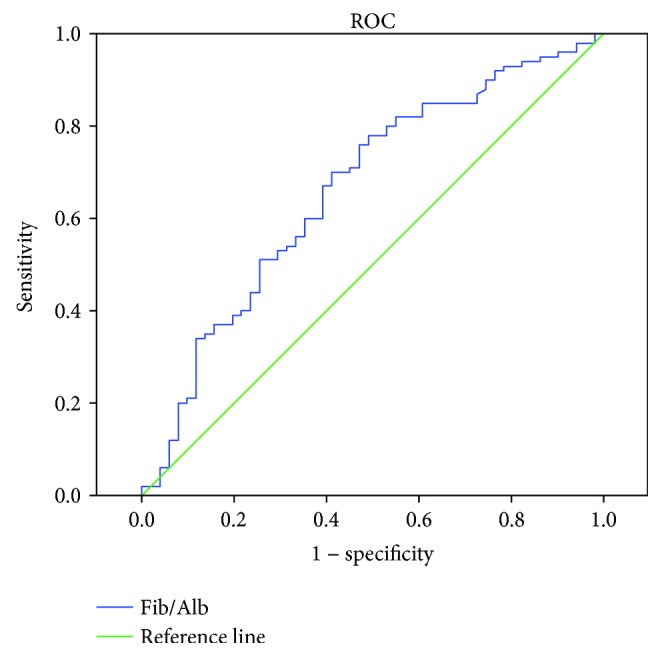
Determination of the cutoff value for the Fib/Alb ratio in patients undergoing curative resection for HCC by ROC analysis.

The cutoff value of the preoperative Fib/Alb ratio was determined using a ROC curve (Figure 1). The Youden index was calculated as (sensitivity + specificity) − 1 for each cutoff point. The highest Youden index for Fib/Alb ratio was 0.279 at 0.062 (sensitivity = 75%, specificity = 52.9%). As a result, 0.062 was selected as the cutoff of fibrinogen/albumin ratio (the area under ROC curve: 0.663, 95% CI: 0.570–0.756, *P* = 0.001).

### 3.3. Relationship between Preoperative Fib/Alb Ratios and the Clinicopathological Characteristics of HCC

According to the Fib/Alb ratio cutoff value determined from the ROC curve, the 151 patients were divided into two groups (Fib/Alb ratio > 0.062, *n* = 101, and Fib/Alb ratio ≤ 0.062, *n* = 50). The relationship between preoperative Fib/Alb ratios and the clinicopathologic variables of patients with HCC was investigated, and the data showed that the preoperative Fib/Alb ratio was associated with microvascular invasion (*P* = 0.022), BCLC stage (*P* = 0.033), and ALBI grade (*P* = 0.003). ALBI grade is a novel score with powerful prognostic value of HCC based on liver function [24]. For the reason that only two patients in our study fell into ALBI grade 3, patients that fell into ALBI grade 2 and ALBI grade 3 were grouped together. No significant relationship was found between the Fib/Alb ratio and other clinicopathologic features (Table 1).

### 3.4. Independent Prognostic Factors of OS and TTR for HCC Patients Receiving Curative Resection

To further identify predictors of postoperative OS and TTR, Fib/Alb ratios, NLR, PLR, ALBI grade, and other clinicopathologic parameters were evaluated via a Cox proportional hazard regression analysis. The selected cutoff value was 2.81 for NLR [9] and 115 for PLR [25]. In the univariate analysis, liver cirrhosis (*P* = 0.025), tumor encapsulation (*P* = 0.041), microvascular invasion (*P* < 0.001), tumor differentiation (*P* < 0.001), BCLC stage (*P* = 0.006), preoperative Fib/Alb ratio (*P* = 0.001), ALBI grade (*P* = 0.006), and NLR (*P* = 0.037) were responsible for the OS. A multivariate regression analysis was performed on all 8 factors that were shown to make a statistically significant difference in OS in the univariate analysis. The results show that liver cirrhosis, microvascular invasion, tumor differentiation, and the Fib/Alb ratio were the independent prognostic predictors of OS (Table 2).

Similarly, the univariate analysis showed that AFP (*P* = 0.044), tumor size (*P* = 0.002), tumor encapsulation (*P* = 0.019), microvascular invasion (*P* < 0.001), tumor differentiation (*P* < 0.001), BCLC stage (*P* = 0.003), preoperative Fib/Alb ratio (*P* = 0.009), NLR (*P* = 0.005), and PLR (*P* = 0.043) were associated with TTR. In a multivariate analysis, microvascular invasion, tumor differentiation, and the Fib/Alb ratio were independent risk factors for TTR (Table 3).

### 3.5. Comparison of the Areas under the Curves for Fib/Alb Ratio, NLR, PLR, ALBI Grade, and Other Clinical Indexes

**Figure 2:**
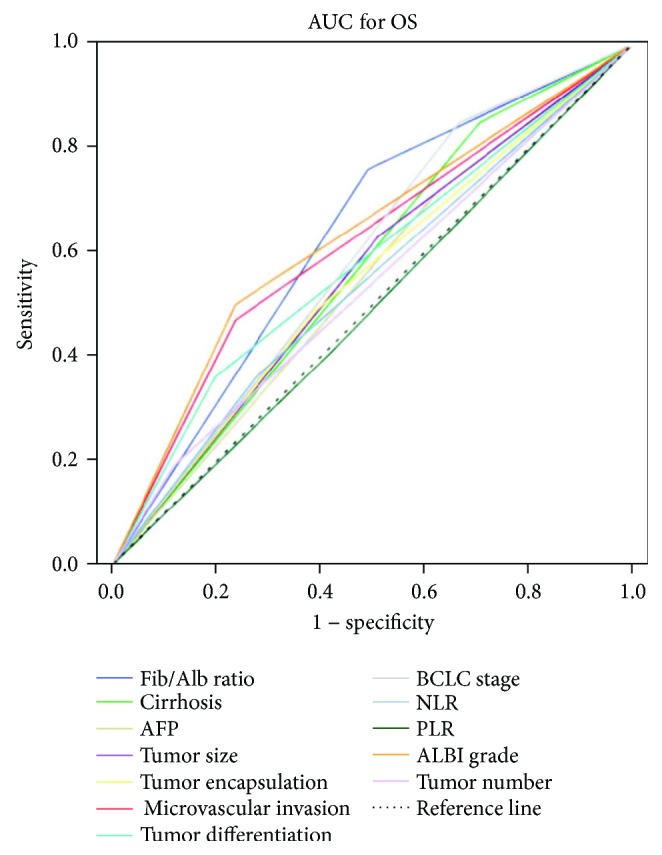
Comparison of the areas under the curves for the Fib/Alb ratio, NLR, PLR, ALBI grade, and other clinical indexes. The discrimination ability of the Fib/Alb ratio, NLR, PLR, ALBI grade, and other clinical indexes was compared by the AUC for OS.

The discrimination ability of the Fib/Alb ratio, two inflammation-based prognostic scores (NLR, PLR), a novel prognostic score of HCC based on liver function (ALBI grade), and other clinical indexes was compared by the AUC (Figure 2). The AUC for the Fib/Alb ratio (dichotomized) was 0.635 (95% CI, 0.539–0.731), which was higher than that for other indexes (cirrhosis, AFP, tumor size, tumor encapsulation, microvascular invasion, tumor differentiation, BCLC stage, NLR, PLR, ALBI grade, and tumor number) for predicting overall survival in HCC patients after curative resection (Table 4).

### 3.6. Association of Fib/Alb Ratios with OS and Recurrence Rates

**Figure 3:**
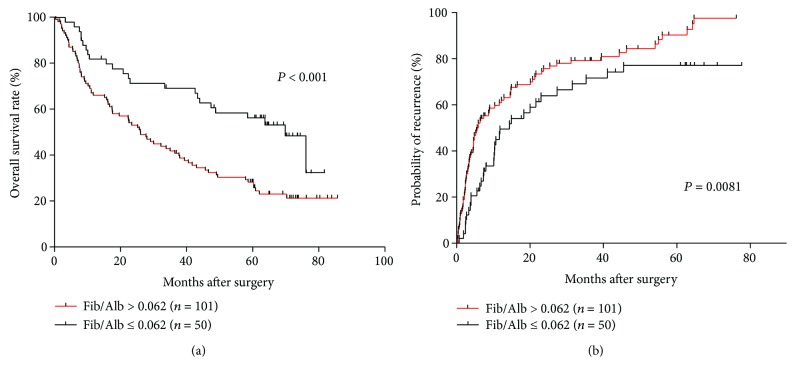
Kaplan–Meier curves of overall survival and recurrence are shown for HCC patients. (a) OS of patients with a Fib/Alb ratio > 0.062 was significantly shorter than that of those with a Fib/Alb ratio ≤ 0.062 (*P* < 0.001, log-rank test). (b) Recurrence rate of patients with a Fib/Alb ratio > 0.062 was significantly higher than that of those with a Fib/Alb ratio ≤ 0.062 (*P* = 0.0081, log-rank test).

To evaluate the prognostic ability of Fib/Alb ratios in predicting OS and recurrence, the 151 HCC patients were divided into two groups: Fib/Alb ratio > 0.062 (*n* = 101) and Fib/Alb ratio ≤ 0.062 (*n* = 50). Cumulative survival and recurrence rates were calculated using a Kaplan–Meier analysis. In terms of OS, patients with a Fib/Alb ratio > 0.062 had lower 1-, 3-, and 5-year OS rates (66.0%, 41.8%, and 28.2% versus 81.9%, 69.3%, and 56.1%, resp., *P* < 0.001) and a shorter OS (median, 26 months versus 69.9 months) compared with patients with a Fib/Alb ratio ≤ 0.062. (Figure 3(a)). In terms of recurrence, patients with a Fib/Alb ratio > 0.062 had higher 1-, 3-, and 5-year recurrence rates (60.9%, 79.2%, and 90.5% versus 49.5%, 69.1%, and 77.1%, resp., *P* = 0.0081) and a shorter TTR (median, 5.9 months versus 14.4 months) compared with patients with a Fib/Alb ratio ≤ 0.062 (Figure 3(b)). Therefore, the result revealed that elevated preoperative Fib/Alb ratios were associated with poor prognoses in HCC patients after curative resection.

### 3.7. Prognostic Significance of the Fib/Alb Ratio in HCC Patients without Microvascular Invasion

**Figure 4:**
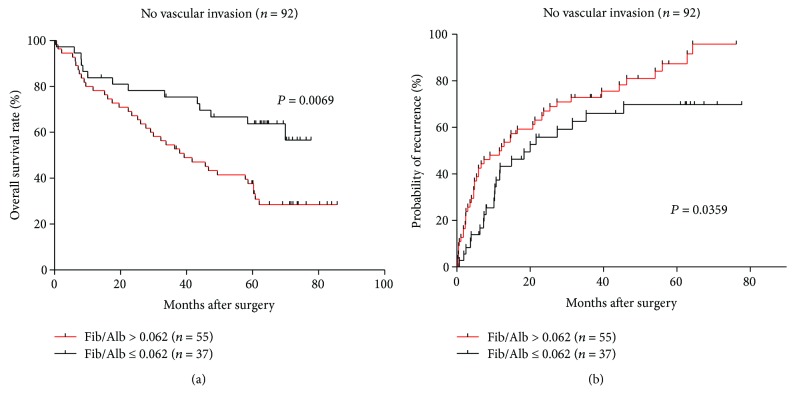
Kaplan–Meier survival curves of HCC patients without microvascular invasion. Fib/Alb ratio > 0.062 was significantly correlated with a shorter OS (a) and higher recurrence rate (b).

We further investigated the prognostic significance of the Fib/Alb ratio in the subgroup of HCC patients without microvascular invasion and found that it was significantly correlated with OS (*P* = 0.0069) and TTR (*P* = 0.0359) (Figures 4(a) and 4(b)). The results reveal that the Fib/Alb ratio was a prognostic factor for OS and TTR in patients without microvascular invasion.

## 4. Discussion

In the 19th century, the observation of leukocytes within tumors by Rudolf Virchow provided the first indication of a possible link between inflammation and cancer. Currently, it is widely accepted that cross talk exists between the inflammatory response and cancer development. Inflammation impacts every step of tumorigenesis, from initiation through tumor promotion and all the way to metastatic progression [7]. In addition, the secretion of growth factors and chemokines by tumors modulates the inflammatory environment and causes a systemic inflammatory response [6, 26]. Numerous data has revealed that the outcome of cancer is influenced not only by tumor-related factors but also by host-related factors, particularly the systemic inflammatory response, which is usually reflected by a variety of biochemical or hematological markers. As a result, several inflammation-based scores, such as NLR, PLR, GPS, and mGPS scoring systems, have been reported to have prognostic value in regard to HCC [9, 12, 13, 25].

Fibrinogen is not only an acute-phase-response protein that reflects systemic inflammatory response but also a vital factor that participates in the maintenance of hemostasis. Both systemic inflammatory response and hemostatic system closely connect with cancer development [7, 27]. Based on its ability to directly bind to members of the vascular endothelial growth factor (VEGF), transforming growth factor-*β* (TGF-*β*), platelet-derived growth factor (PDGF), and fibroblast growth factor (FGF) families, fibrinogen has been reported to play a critical role in cell proliferation, the epithelial-to-mesenchymal transition (EMT), angiogenesis, and the hematogenous metastasis of tumor cells [28–30]. In addition, some clinical studies have shown that high pretreatment plasma fibrinogen levels are related to poorer prognoses in a variety of tumors [16, 31–33]. Perisanidis et al. proposed that adjunct treatments lowering plasma fibrinogen concentration may hold promise for prolonging survival in patients with solid tumors [34]. What is more, it is suggested that therapies targeting fibrinogen-dependent interactions may make a positive contribution to the treatment of some kinds of malignancies [35, 36].

Albumin is the most abundant plasma protein accounting for about 50% of the total protein content [37]. The low concentration of albumin may reflect bad nutritional status and performance status. Malnutrition may weaken the immune system and negatively impact the prognosis of cancer patients [38]. Furthermore, albumin is also an important factor that participates in the systemic inflammatory response. The prognostic value of pretreatment albumin has been reported in various human malignancies, including renal cell carcinoma [39], head and neck cancers [40], non-small cell lung cancer [41], ovarian cancer [42], and adenocarcinoma of the gastric cardia [43]. In addition, serum albumin is one of the components of the Child–Pugh classification system that reflects liver function. It has also been reported that hypoalbuminemia is an independent prognostic factor associated with poor outcomes in patients with HCC [44].

It was reported that the fibrinogen/albumin ratio was a novel blood tool of cancer prognosis in esophageal squamous cell carcinoma recently [21]. What is more, Kinoshita et al. found the prognostic value of C-reactive protein (CRP)/albumin ratio in patients with HCC [11]. Similar to CRP, fibrinogen belongs to the positive acute-phase-response proteins, characterized by its elevation during systemic inflammation [45]. It has been reported that there was a strong positive correlation between fibrinogen and CRP levels in thoracic malignancies [14, 46]. Inspired by these, we try to investigate whether fibrinogen/albumin ratio has a prognostic value in HCC patients after curative resection.

We report here for the first time, to the best of our knowledge, that the Fib/Alb ratio is an independent prognostic factor for HCC following curative hepatectomy. Hepatitis B or C infections and alcohol consumption are major risk factors of HCC. Patients with HCC are typically characterized by impaired liver function due to concomitant intrahepatic chronic inflammation and liver cirrhosis. Since fibrinogen, albumin, and CRP are synthesized by hepatocytes, impaired liver function will influence the accuracy of making assessments based on fibrinogen or albumin alone and of the GPS and mGPS scoring systems, which score serum CRP and albumin levels separately, in predicting a prognosis for patients with HCC. In contrast, the Fib/Alb ratio reflects the ratio of the fibrinogen and albumin levels, thus reducing the influence of poor liver function. It may reflect the association between cancer progression and the host's inflammatory environments more effectively in patients with HCC. We also compare the Fib/Alb ratio with a novel prognostic score of HCC based on liver function (ALBI grade). The Fib/Alb ratio was associated with the ALBI grade (*P* = 0.003, Table 1). The AUC for OS of the Fib/Alb ratio was 0.635, which was comparable to the ALBI grade (0.632) (Figure 2, Table 4). Meanwhile, Fib/Alb ratio measurements are based on standard laboratory measurements of fibrinogen and albumin, which are routinely performed in clinical practice. Hence, the Fib/Alb ratio is a promising and convenient biomarker for predicting HCC prognoses.

In our study, we first determined that the optimal cutoff value of the Fib/Alb ratio was 0.062 using a ROC curve analysis. Then, the relationship between preoperative Fib/Alb ratios and the clinicopathological variables of HCC patients was investigated, and the data showed that elevated Fib/Alb ratio was positively associated with microvascular invasion, BCLC stage, and ALBI grade. Furthermore, a multivariate Cox regression analysis revealed that microvascular invasion, tumor differentiation, and the Fib/Alb ratio were independent prognostic predictors of OS and TTR. Moreover, the ROC analysis demonstrated that the Fib/Alb ratio (dichotomized) had a higher AUC value than NLR, PLR, ALBI grade, and other clinical indexes for predicting OS. It suggested that the Fib/Alb ratio was comparable to that of other established prognostic scores (NLR, PLR, and ALBI grade) in terms of its prognostic ability. The prognostic value of the Fib/Alb ratio was further analyzed, and we found that patients with a Fib/Alb > 0.062 had lower 1-, 3-, and 5-year OS rates and shorter OS times, as well as higher 1-, 3-, and 5-year recurrence rates and shorter TTRs compared with patients with a Fib/Alb ≤ 0.062. Moreover, the Fib/Alb ratio had significant prognostic value for both OS and TTR in patients without microvascular invasion.

Predicting recurrence following curative resection is critical for the management of HCC. Up to now, there are still no highly reliable and convenient predictive biomarkers for the recurrence of HCC. Although AFP is widely used, its predictive effectiveness is poor as 30% to 40% of patients with HCC have normal serum AFP levels after surgery [47]. Encouragingly, our study provides the Fib/Alb ratio as an alternative option for predicting recurrence. Furthermore, microvascular invasion was recognized as an independent predictor of early recurrence in HCC patients who underwent curative surgery [48]. In clinical practice, it is difficult to predict which individuals will experience tumor relapse after surgical treatment in the absence of microvascular invasion. Finding a predictor to discriminate at-risk patients from this subpopulation is therefore important. In the current study, after stratifying the patient cohort according to the presence and absence of microvascular invasion, we found that the prognostic significance of the Fib/Alb ratio was still strong in HCC patients without microvascular invasion. Consequently, patients with higher Fib/Alb ratios in the subgroup of HCC patients without microvascular invasion may require a closer follow-up since they are more likely to suffer from tumor recurrence. If recurrence is predicted early and prevented in a timely manner, these patients will achieve a favorable outcome.

There are several limitations to the current study. First, it was a retrospective, single-center study with a small sample size, and a well-designed, prospective study with a larger number of patients is needed. Second, owing to the relatively small number of patients, we did not divide the patients into a training cohort and a test cohort for statistical validation. Third, many other factors affect Fib/Alb ratios, such as acute undetected infections and hematological diseases, which affect the accuracy of prognostic predictions based on this ratio. Fourth, the different postoperative treatments that patients received were not included into the prognostic factor evaluation. Furthermore, many patients received multiple treatment measures due to tumor recurrence during their follow-up periods, which affects OS. Fifth, as 87.4% of the patients in our study were hepatitis B virus positive, the prognostic significance of the Fib/Alb ratio needs to be validated in HCC patients from the United States and Europe since hepatitis C is the most common risk factor for developing HCC in these geographic areas.

In conclusion, we demonstrated for the first time that the preoperative Fib/Alb ratio is a useful prognostic factor for assessing HCC patients following curative resection, particularly for patients without microvascular invasion. Its utility, convenience, and low cost make the Fib/Alb ratio a promising serum biomarker for predicting HCC prognoses and may support the therapeutic decisionmaking process for HCC patients in the future.

## Figures and Tables

**Table 1:** Relationship between preoperative Fib/Alb ratio and clinicopathological characteristics.

Clinical and pathologic indexes	Cases *n* = 151	Fib/Alb ratio		*P* value
		>0.062 (*n* = 101)	≤0.062 (*n* = 50)	
Gender				
Male	128	89	39	0.147
Female	23	12	11	
Age (years)				
>50	76	49	27	0.605
≤50	75	52	23	
HBs antigen				
(+)	132	88	44	1.00
(−)	19	13	6	
Cirrhosis				
Yes	121	82	39	0.668
No	30	19	11	
AFP (*μ*g/L)				
>20	104	69	35	1.00
≤20	47	32	15	
Tumor size (cm)				
>5	89	65	24	0.078
≤5	62	36	26	
Tumor encapsulation				
Complete	69	46	23	1.00
None	82	55	27	
Microvascular invasion				
Yes	59	46	13	0.022^∗^
No	92	55	37	
Tumor differentiation				
I-II	105	67	38	0.263
III-IV	46	34	12	
BCLC stage				
0 + A	32	16	16	0.033^∗^
B + C	119	85	34	
ALBI grade				
1	89	51	38	0.003^∗^
2 + 3	62	50	12	

^∗^ *P* < 0.05.

**Table 2:** Univariate and multivariate Cox regression analyses of prognostic factors for overall survival (OS).

Variables	Univariate analyses		Multivariate analyses	
	HR (95% CI)	*P* value	HR (95% CI)	*P* value
Gender				
Male	0.779 (0.442–1.374)	0.389		
Female				
Age (years)				
>50	0.864 (0.583–1.279)	0.465		
≤50				
HBs antigen				
(+)	0.913 (0.519–1.607)	0.753		
(−)				
Cirrhosis				
Yes	1.876 (1.082–3.250)	0.025^∗^	1.994 (1.145–3.475)	0.015^∗^
No				
AFP (*μ*g/L)				
>20	1.458 (0.942–2.258)	0.091		
≤20				
Tumor size (cm)				
>5	1.444 (0.961–2.169)	0.077		
≤5				
Tumor encapsulation				
Complete	1.509 (1.018–2.237)	0.041^∗^		
None				
Microvascular invasion				
Yes	2.236 (1.503–3.326)	<0.001^∗^	1.552 (1.009–2.389)	0.046^∗^
No				
Tumor differentiation				
I-II	2.539 (1.676–3.846)	<0.001^∗^	2.189 (1.394–3.437)	0.001^∗^
III-IV				
BCLC stage				
0 + A	2.181 (1.256–3.789)	0.006^∗^		
B + C				
Fib/Alb ratio				
>0.062	2.146 (1.353–3.404)	0.001^∗^	2.015 (1.266–3.207)	0.003^∗^
≤0.062				
ALBI grade				
1	1.751 (1.178–2.604)	0.006^∗^		
2 + 3				
NLR				
≥2.81	1.546 (1.027–2.327)	0.037^∗^		
<2.81				
PLR				
≥115	1.221 (0.817–1.823)	0.330		
<115				

HR: hazard ratio; 95% CI: 95% confidence interval; BCLC: Barcelona Clinic Liver Cancer. Cox regression analysis, ^∗^ *P* < 0.05.

**Table 3:** Univariate and multivariate Cox regression analyses of prognostic factors for time to recurrence (TTR).

Variables	Univariate analyses		Multivariate analyses	
	HR (95% CI)	*P* value	HR (95% CI)	*P* value
Gender				
Male	1.319 (0.806–2.160)	0.271		
Female				
Age (years)				
>50	0.705 (0.49–1.014)	0.06		
≤50				
HBs antigen				
(+)	0.728 (0.440–1.203)	0.215		
(−)				
Cirrhosis				
Yes	1.160 (0.740–1.819)	0.517		
No				
AFP (*μ*g/L)				
>20	1.510 (1.012–2.254)	0.044^∗^		
≤20				
Tumor size (cm)				
>5	1.822 (1.245–2.666)	0.002^∗^		
≤5				
Tumor encapsulation				
Complete	1.556 (1.076–2.248)	0.019^∗^		
None				
Microvascular invasion				
Yes	2.104 (1.442–3.072)	<0.001^∗^	1.584 (1.037–2.421)	0.033^∗^
No				
Tumor differentiation				
I-II	2.326 (1.529–3.539)	<0.001^∗^	1.718 (1.081–2.730)	0.022^∗^
III-IV				
BCLC stage				
0 + A	2.034 (1.269–3.261)	0.003^∗^		
B + C				
Fib/Alb ratio				
>0.062	1.704 (1.142–2.543)	0.009^∗^	1.555 (1.031–2.346)	0.035^∗^
≤0.062				
ALBI grade				
1	1.131 (0.783–1.632)	0.512		
2 + 3				
NLR				
≥2.81	1.723 (1.177–2.523)	0.005^∗^		
<2.81				
PLR				
≥115	1.466 (1.013–2.121)	0.043^∗^		
<115				

HR: hazard ratio; 95% CI: 95% confidence interval; BCLC: Barcelona Clinic Liver Cancer. Cox regression analysis, ^∗^ *P* < 0.05.

**Table 4:** Comparison of the areas under the curves for Fib/Alb ratio, NLR, PLR, ALBI grade, and other clinical indexes.

Variables	AUC	95% CI	*P* value
Fib/Alb ratio (dichotomized)	0.635	0.539–0.731	0.007^∗^
Cirrhosis (dichotomized)	0.572	0.473–0.671	0.148
AFP (dichotomized)	0.546	0.448–0.645	0.353
Tumor size (dichotomized)	0.56	0.463–0.658	0.228
Tumor encapsulation (dichotomized)	0.549	0.452–0.646	0.326
Microvascular invasion (dichotomized)	0.617	0.525–0.710	0.019^∗^
Tumor differentiation (dichotomized)	0.582	0.488–0.676	0.1
BCLC stage (dichotomized)	0.592	0.493–0.691	0.066
NLR (dichotomized)	0.543	0.446–0.639	0.391
PLR (dichotomized)	0.494	0.396–0.592	0.906
ALBI grade (dichotomized)	0.632	0.540–0.724	0.008^∗^
Tumor number (dichotomized)	0.536	0.440–0.632	0.468

^∗^ *P* < 0.05.

## Supplementary Materials

Supplementary Materials Supplementary Figure 1: the ROC analysis of NLR (continuous), PLR (continuous), and ALBI grade (dichotomized) in 151 HCC patients who underwent curative resection.
