# Supplementary figures and images for: A Novel Inflammation-Based Prognostic Score: The Fibrinogen/Albumin Ratio Predicts Prognoses of Patients after Curative Resection for Hepatocellular Carcinoma
Source: J Immunol Res. 2018 May 22;2018:4925498. doi: 10.1155/2018/4925498 (PMC6031154; doi:10.1155/2018/4925498)

## Slide 1
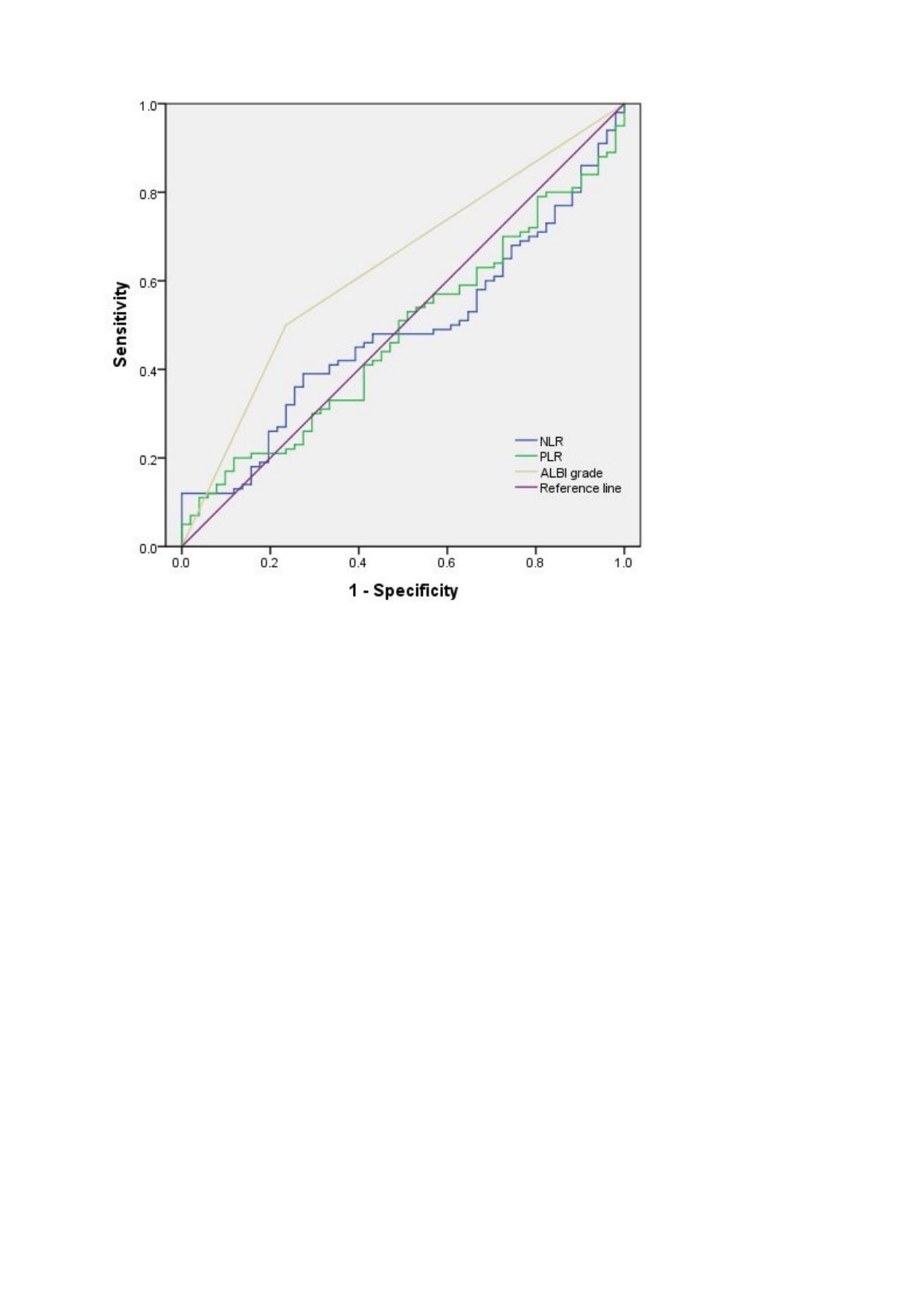

Supplement: Supplementary Materials — Supplementary Figure 1: the ROC analysis of NLR (continuous), PLR (continuous), and ALBI grade (dichotomized) in 151 HCC patients who underwent curative resection. [file 4925498.f1.ppt]
